# Sociodemographic Variables and Psychological Distress of Moroccan College Students

**DOI:** 10.5964/ejop.11689

**Published:** 2024-11-29

**Authors:** Hatim Ben Ayad, Adil Najdi, Meftaha Senhaji

**Affiliations:** 1 UAE/U08FS, FS, Abdelmalek Essaâdi University, Tetouan, Morocco; 2 Faculty of Medicine and Pharmacy, Abdelmalek Essaâdi University, Tetouan, Morocco; Università Cattolica del Sacro Cuore, Milan, Italy

**Keywords:** mental health, psychological distress, sociodemographic variables, Moroccan students, college students

## Abstract

Because college students are an important category of the population highly vulnerable to mental health problems, this study aims to investigate the sociodemographic variables associated with the increase in psychological distress levels among Moroccan college students. Participants (*N* = 1147; mean age 20.00, *SD* = 2.6; 703 females and 444 males) completed a survey, which included a sociodemographic questionnaire and the Arabic version of the Brief Symptoms Inventory (BSI). Non-parametric tests were conducted to explore the data. Non-parametric tests revealed that being female, having a physical illness, experiencing depression and anxiety, having sleep problems, and living with only their mother or with a family member other than their parents are associated with a significant increase in the level of psychological distress. In summary, specific sociodemographic factors exert a notable influence on the psychological distress levels experienced by college students. Consequently, it is imperative to intensify research endeavors aimed at delving into the intricacies of college students’ mental health and its correlated ramifications.

College student populations are particularly vulnerable to the development of mental health problems (Liu et al., 2019; Mortier et al., 2018). Their experiences in college are marked by heightened stress due to the burden of responsibility, independence, and academic demands, making them more susceptible to psychological distress and the onset of mental disorders (Liu et al., 2019; Pedrelli et al., 2015). Despite being recognized as a pivotal contributor to the country’s future wealth and economy (Knapp & Wong, 2020), there is a notable dearth of research on the mental health of Moroccan college students and its associated factors.

The concept of psychological distress is known to be vague and hard to define. However, it is clear that it serves as a significant indicator of psychological suffering, often presenting as symptoms of depression and anxiety, accompanied by specific personality traits and behavioral issues (Drapeau et al., 2012; Staneva et al., 2015). Consequently, symptoms of anxiety and depression are frequently assessed through the framework of psychological distress (Chiu et al., 2018; Copeland et al., 2014; Gore et al., 2011), alongside of other somatic symptoms and behavioral problems (Drapeau et al., 2012).

One of the significant reasons to draw attention to college students is the alarming prevalence of mental health problems within this demographic worldwide (Auerbach et al., 2016; Auerbach et al., 2018). Existing studies on Moroccan college students underscore the substantial prevalence of mental distress, particularly among medical students. A study involving 358 medical students, predominantly females (238 individuals), at the Faculty of Medicine in Marrakech, employing the General Health Questionnaire (GHQ) (Goldberg, 1972), revealed that over 66% of participants exhibited psychological distress, scoring above 4 points on the GHQ (Benali & Chichou, 2020). Moreover, another study conducted on 637 medical students, with a majority being females (66%), at the Faculty of Medicine and Pharmacy in Rabat, utilizing the French version of the GHQ (Salama-Younes et al., 2009), demonstrated that 298 participants (46.9%) experienced psychological distress, surpassing the four-point threshold (Lemtiri Chelieh et al., 2019).

Notably, among the critical factors interlinked with psychological distress, stressful life events assume prominence, particularly within the college environment (Liu et al., 2019). Likewise, psychological distress is associated with various sociodemographic factors. Gender, for instance, has been identified as a significant contributor to psychological distress (Drapeau et al., 2012). Mental distress levels differ based on gender, with females generally reporting higher levels of psychological distress than males (Boyd et al., 2015; Van Droogenbroeck et al., 2018). In response to stress, traumatic events, or other risk factors, gender differences in mental health problems are reflected in different types of mental disorders experienced; males are more likely to suffer from substance use disorders and antisocial behavior, while females are more prone to depression and anxiety (Rosenfield & Mouzon, 2013). Therefore, females are expected to show higher levels of psychological distress since they are more likely to suffer from anxiety and depression symptoms than males.

Moreover, living in a college environment may require changes in living arrangements, potentially leading to increased psychological distress levels (Agerup et al., 2015). For example, living with roommates and reduced parental control may result in unhealthy behaviors such as alcohol consumption and smoking, both negatively associated with psychological distress levels (Deasy et al., 2015; Lorant et al., 2013). Additionally, separation from parents may result in the absence of parental support to cope with college stress, leading to increased psychological distress (Reeve et al., 2013). Moreover, Moroccan college students who are not living with their parents report more self-efficacy (Otmane et al., 2020), and self-efficacy is negatively associated with the psychological distress (Grøtan et al., 2019; Saleh et al., 2017). Therefore, investigating living arrangements effect on the psychological distress levels of Moroccan college students is very important.

In addition, college students are typically responsible for their well-being, for the first time for freshmen, including their physical health (Ridner et al., 2016). Therefore, their physical health may be vulnerable to unhealthy behaviors or a lack of healthcare-seeking behavior. Additionally, the stressful college environment can lead to fatigue (Kizhakkeveettil et al., 2017), and both fatigue and stress are linked to the physical well-being of college students (Jacob et al., 2013). Thus, college students suffering from physical illnesses are more likely to report more psychological distress than healthy individuals (de Rooij et al., 2021; Saifullah et al., 2020)

Lastly, poor sleep quality has been found to be associated with an increased risk of developing mental health problems (Chu & Richdale, 2009). In fact, sleep problems are associated with increased psychological distress as they contribute to elevated depression and anxiety symptoms (Ghrouz et al., 2019). Furthermore, college students often report significantly low sleep quality compared to other demographics (Carter et al., 2017; Dinis & Bragança, 2018), with stress considered one of the main factors responsible for low sleep quality (Almojali et al., 2017).

## Study Aims

Given the heightened susceptibility of college students to psychological distress and the lack of research on Moroccan college students’ mental health, this study aims to investigate the sociodemographic factors associated with psychological distress among Moroccan college students.

## Method

### Participants and Procedure

The sample strategy used for this study was stratified cluster sampling. At first, the sampling was based on three levels: 1) the city, 2) the type of establishment (open access, i.e., institutions with unlimited number of seats vs. regulated access, i.e., institutions with limited number of seats), and 3) study level (i.e., undergraduate students of the first year, undergraduate students of the second year, and undergraduate of the last year and graduate students). For each institution, we defined a two-stage cluster in which the precise sample size was randomly selected as a function of the institution’s number of students.

The numbers of the total samples and the subsamples from each institution were calculated using Epi-Info 7 software’s Stat Calc application, with the following parameters:

- Source Population size: 100, 828 subjects (students of Abdelmalek Essaâdi University).
- Maximum tolerated error margin: fixed at 3%.
- Sampling method: stratified cluster sampling with three layers.
- Number of layers: 9 layers.
- Expected frequency of the parameter to be estimated: 50% by caution (max size with 50%).

This study aimed for a medium effect size, denoted by Cohen’s *d* = 0.5, indicating a moderate difference between groups. Setting the significance level (α) at .05, the study aimed for a power of .80, signifying an 80% chance of detecting the hypothesized effect if it genuinely exists. With these parameters in mind, the power analysis was conducted using a two-sample *t*-test. The goal was to achieve a power of .80, which is widely considered acceptable in psychological research.

Considering these criteria and the complex nature of the study, including stratified cluster sampling with nine layers and a maximum tolerated error margin of 3%, the power analysis revealed that a total sample size of 1060 participants would be necessary to achieve the desired power level. To account for potential refusals and non-responses, the sample size was increased by 10%, resulting in a final sample size of 1200 students.

Of 1200 graduate and undergraduate students asked to participate in the study, only 1147 questionnaires were retained after excluding uncompleted questionnaires. The sample was composed of students of Abdelmalek Essaâdi University that includes eight establishments; five faculties and three institutions.

Most of the participants were females (*n* = 703, 61.1%), and 444 of the participants (38.6%) were males. The mean age of the participants was 20.00 (*SD* = 2.6) years (range: 17 to 49 years). Most of the participants were single (*n* = 1021, 88.8%), 94 (8.2%) were in relationships, 13 (1.1%) were no longer in a relationship, and 22 (1.9%) participants did not answer the question about their marital status. Finally, 407 (35.7%) of the participants were undergraduate students in the third year and graduate students, 369 (32.1%) of the participants were undergraduate students of the second year, and 371 (32.3%) were undergraduate students of the first year.

### Instruments

*The Brief Symptom Inventory* is a self-reported instrument that assesses psychological symptoms across nine dimensions: somatization, obsession-compulsion, interpersonal sensitivity, depression, anxiety, hostility, phobic anxiety, paranoid ideation, and psychoticism. It comprises 53 items rated on a 5-point Likert scale, with responses ranging from 0 (Not at all) to 4 (Extremely) (Derogatis & Melisaratos, 1983). The Cronbach’s alpha coefficient results for the nine dimensions ranged from .70 to .89, indicating adequate internal consistency (Broday & Mason, 1991; Pereda et al., 2007). The Arabic version of the BSI used in this study has demonstrated satisfactory internal consistency, with values ranging from .70 to .83 across the nine dimensions (Abdallah, 1998).

Furthermore, in Zouini et al.’s (2019) study involving a sample of Moroccan high school students, the internal consistency of the nine dimensions of the BSI ranged from 0.71 to 0.85. In this study, Cronbach’s alpha coefficients ranged from .69 to .81. The Positive Symptom Total (PST), which counts the number of symptoms that the respondent has experienced, was selected to assess participants’ current level of psychological distress and showed excellent internal consistency (α = .94).

### Statistical Analysis

Since the data did not follow normal distribution (Skewness = .074 and Kurtosis = -.089), non-parametric tests were performed to compare different groups scoring on BSI. All statistical analyses were performed using the Statistical Package for the Social Sciences (SPSS) 21.0 (IBM) software for Windows.

## Results

### Sociodemographic Characteristics of the Study Sample

The study sample was composed of 61.1% females (*n* = 703) and 38.6% males (*n* = 444). The living arrangements of the participants were as follows: 70.2% (*n* = 807) were living with their parents, 7.8% (*n* = 90) were living with their mother, 1.5% (*n* = 17) were living with their father, 11.7% (*n* = 134) were living alone, and 6.4% (*n* = 74) were living with another family member. Additionally, 16.6% (*n* = 191) reported consulting the hospital due to a physical illness, 3.7% (*n* = 42) reported taking medication for depression, 7.6% (*n* = 87) reported taking medication for anxiety, and 4.6% (*n* = 53) reported taking medication for sleep (Table 1).

**Table 1:** Sociodemographic Characteristics of the Study Sample

Sociodemographic Characteristic		*N* (%)
Gender	Male	444 (38.6%)
	Female	703 (61.1%)
Living Arrangements	With parents	807 (70.2%)
	With mother only	90 (7.8%)
	With father only	17 (1.5%)
	Alone	134 (11.7%)
	Other family member	74 (6.4%)
Consulting hospital for physical problem in the past month	Yes	191 (16.6%)
	No	912 (79.3%)
Taking medication for anxiety in the past month	Yes	87 (7.6%)
	No	1004 (87.3%)
Taking medication for sleep problems in the past month	Yes	53 (4.6%)
	No	1064 (92.5%)
Taking medication for depression problems in the past month	Yes	42 (3.7%)
	No	1042 (90.6%)

### Sociodemographic Variables Association With Scoring on BSI

The comparison of Positive Symptom Total (PST) scores on the BSI among different groups revealed significant differences: between male participants and female participants (*Z* = -5.77, *p* < .001), between participants who reported consulting a hospital for a physical problem during the past month and those who did not (*Z* = -5.946, *p* < .001), between participants who reported taking medication for depression in the past month and those who did not (*Z* = -3.776, *p* < .001), between participants who reported taking medication for anxiety in the past month and those who did not (*Z* = -5.246, *p* < .001), and between participants who reported taking medication for sleep troubles in the past month and those who did not (*Z* = -3.992, *p* < .001). Additionally, a significant difference was found when comparing BSI-PST scores of participants who reported different living arrangements $\chi^{2}$(4) = 13.47, *p* = .008 (Table 2).

**Table 2:** PST Scores of Different Groups

Sociodemographic Variable		Mean (*n*)	*SD*	Z	*P*
Gender	Male	64.12 (370)	31.33	-5.77	.000
	Female	76.03 (603)	31.10		
Consulting hospital for a physical problem	Yes	85.45 (171)	33.38	-5.946	.000
	No	68.52 (802)	30.54		
Taking medication of depression in the past month	Yes	87.67 (30)	26.09	-3.776	.000
	No	70.99 (943)	31.74		
Taking medication of anxiety in the past month	Yes	93.20 (67)	30.41	-5.246	.000
	No	69.90 (906)	31.22		
Taking medication of sleep in the past month	Yes	93.06 (46)	32.88	-3.992	.000
	No	70.43 (927)	31.28		
Living arrangements	With parents	70.50 (690)	32.02	χ2 = 13.467	.008
	With mother alone	76.83 (81)	30.9		
	With father alone	69.35 (14)	32.7		
	Alone	71.16 (128)	30.21		
	With another family member	80.5 (60)	29.83		

*Note*. *P* is significant at the 5% level.

## Discussion

In this study, female participants scored significantly higher than their male counterparts. In fact, females exhibit heightened sensitivity to stress, potentially elevating the susceptibility to anxiety symptoms (Parker & Brotchie, 2010), leading to an increased psychological distress than males. Additionally, disparities in social activities and character traits exist between genders (Rosenfield & Mouzon, 2013). For instance, a study involving 2,588 individuals from the Spanish general population unveiled that the elevated psychological distress levels observed in females compared to males can be attributed to the prevalent use of emotion-focused coping mechanisms among females, a strategy associated with heightened psychological distress (Matud et al., 2015). Moreover, gender-based differences in social status, equity, and opportunities correlate with adverse mental health outcomes for females (Van Droogenbroeck et al., 2018; WHO, 2000).

In terms of physical illness, the results of this study revealed that participants who reported consulting a hospital due to a physical illness in the month before participating in this study had significantly higher PST scores than participants who did not report a physical problem. This association can be explained by the fact that physical illness has been associated with an increase in feelings of stigma and loneliness (Arslan, 2021; Gamwell et al., 2018), and stigma and loneliness have been found to be associated with an increase in psychological distress symptoms (McIntyre et al., 2018; Menec et al., 2020).

Moreover, participants who reported taking medication for anxiety and depression scored significantly higher than participants who did not. Participants who reported taking medication for depression and anxiety during the previous month of data collection suffer from depression and anxiety symptoms, and these later are highly linked to the psychological distress (Copeland et al., 2014; Gore et al., 2011), which supports the results of this study. Additionally, participants who reported taking medication for sleep disturbances during the past month scored significantly higher than participants who did not. Taking medication for sleep problems means that the concerned participants are actually suffering from sleep disturbance. One of the possible explanations of the association between sleep problems and increased psychological distress is stress. This latter was found to increase significantly among college students who reported poor sleep quality (Almojali et al., 2017). Also, sleep problems were found directly associated with an increase in anxiety and depression symptoms which lead to an increased psychological distress (Chiu et al., 2018; Ghrouz et al., 2019).

Lastly, this study’s findings indicate that participants who reported living solely with their mothers and those residing with a family member other than their parents scored significantly higher in PST compared to participants with different living arrangements. This result suggests that separation from parents or the absence of a father is linked to increased psychological distress. The heightened psychological distress in cases of parental separation can be elucidated by the lack of parental support to cope with stress (Reeve et al., 2013). Another possible explanation lies in participants’ potential discomfort when living with a family member other than their parents, fostering dissatisfaction with the living arrangement and, consequently, elevated psychological distress (Kumaraswamy, 2013). Regarding the increased psychological distress among students living solely with their mothers, this result might be attributed to the absence of a father, whether due to death or family breakdown, a factor associated with mental health problems (Amato, 2001; Patton et al., 2014).

## Conclusion

College students undergo a critical phase marked by an increased susceptibility to developing mental health concerns. This study’s findings indicate noteworthy trends: females tend to report significantly higher levels of psychological distress compared to males. Additionally, a correlation exists between physical illness and heightened psychological distress symptoms, as well as a link between sleep disturbances and increased psychological distress levels among college students. These revelations align consistently with findings from various studies (Boyd et al., 2015; Chu & Richdale, 2009; de Rooij et al., 2021; Dwyer-Lindgren et al., 2017; Kawada et al., 2011; Redeker et al., 2010; Saifullah et al., 2020; Wiklund et al., 2012). Furthermore, the study indicates that living arrangements and experiences of anxiety and depression can act as pivotal risk factors contributing to the exacerbation of psychological distress levels among college students. Consequently, further efforts are needed among this demographic to deeply investigate mental health of college students and its outcomes on their academic performance and social life.

One potential avenue of the efforts involves conducting further research to develop requisite tools for assessing mental health among college students. Additionally, intervention initiatives can be crafted around awareness campaigns concerning mental health and disorders. These efforts could also include coaching sessions designed to equip students with effective stress-coping mechanisms. Moreover, integrating mental health services within college faculties and institutions could provide essential support to students in need.

### Strengths and Limitations

The large sample size representing the college students of Abdelmalek Essaâdi University that has faculties and institutions in five different cities, along with the sociodemographic variables found associated with increased levels of psychological distress, make this study a considerable step forward in mental health-related research among college students in Morocco.

The first limitation of this study is its cross-sectional nature, which prevents the investigation of changes in participants’ answers over a specific period. Secondly, the absence of a mental disorders screening tool limits the in-depth exploration of the study’s results. Thirdly, the study is limited by participants who reported taking medication, which could potentially influence their scores on the BSI. Lastly, the study did not collect information on the participants’ socio-economic situations and their families, restricting the exploration of the study’s results.
